# Erratum: Molecular and cellular signatures underlying superior immunity against *Bordetella pertussis* upon pulmonary vaccination

**DOI:** 10.1038/mi.2017.110

**Published:** 2018-02-07

**Authors:** R H M Raeven, J Brummelman, J L A Pennings, L van der Maas, K Helm, W Tilstra, A van der Ark, A Sloots, P van der Ley, W van Eden, W Jiskoot, E van Riet, C A C M van Els, G F A Kersten, W G H Han, B Metz

**Keywords:** Bacterial infection, Mucosal immunology, Signal transduction, Vaccines

**Correction to:** *Mucosal Immunology* advance online publication, 20 September 2017; doi: 10.1038/mi.2017.81

This article was originally published under NPG’s License to Publish, but has now been made available under a CC BY 4.0 license. The PDF and HTML versions of the paper have been modified accordingly.

